# Analysis of Auditory Measures in Normal Hearing Young Male Adult Cigarette Smokers Using Multiple Variable Selection Methods with Predictive Validation Assessments

**DOI:** 10.1155/2009/745151

**Published:** 2009-11-22

**Authors:** Kamakshi V. Gopal, Richard Herrington, Jacquelin Pearce

**Affiliations:** ^1^Department of Speech and Hearing Sciences, University of North Texas, Denton, TX 76203, USA; ^2^Academic Computing Services, University of North Texas, Denton, TX 76203, USA

## Abstract

Studies have shown that cigarette smoking is a risk factor for hearing loss; however, no information is available on auditory preclinical indicators in young chronic cigarette smokers. Cigarette smoking involves exposure to many harmful chemicals including carbon monoxide (CO). In this study, the CO level in 16 young normal hearing male chronic smokers was measured with a CO monitor, and was used as the outcome measure. Subjects were administered a battery of audiological tests that included behavioral and electrophysiologic measures. The goal was to investigate which auditory test measures can be used as potential predictors of the outcome measure. Using ordinary least squares estimation procedures with best-subsets selection and bootstrapped stepwise variable selection procedures, an optimal predictive multiple linear regression model was selected. Results of this approach indicated that auditory brainstem response peak V amplitudes and distortion product otoacoustic emissions had the highest predictive value and accounted for most of the variability.

## 1. Introduction

Recent years have seen a sharp increase in the number of young adults that regularly smoke tobacco cigarettes. According to the Centers for Disease Control and Prevention (CDC), the highest rate of smoking in the US is among 18–24 year-old adults, and there has been a national health objective to reduce the prevalence of smokers in that age group [1]. The risk of developing smoking-related diseases, such as cancer, heart disease, stroke, and respiratory illnesses, has been extensively researched, but relatively little data exists on the specific auditory mechanisms affected by smoking. The effect of cigarette smoking is related to total lifetime exposure to cigarette smoke, which includes the number of cigarettes a person smokes each day, the age at which smoking began, the number of years a person has smoked, and the smoker's exposure to secondhand smoke.

The smoke from a cigarette containing more than 4000 chemicals has over 4500 complex chemicals in them including carbon monoxide (CO), nicotine, and carbon dioxide. Current methods to evaluate smoking status include blood or urine tests that measure plasma nicotine, or the less invasive breath CO concentration measurement [2–5]. Of these tests, breath CO concentration measurement provides immediate assessment without further need for laboratory tests. Many portable breath CO monitors are currently on the market, and when used in conjunction with a questionnaire, have been found to provide a rapid and accurate assessment of exposure to cigarette smoke [5, 6]. Dolcini et al. [4] found the specificity of CO testing in relation to smoking status to be 98%. After cigarette smoke inhalation, CO displaces the oxygen in red blood cells forming carboxyhaemoglobin (COHb). In the form of COHb, the cigarette-derived CO has a conservative half-life of approximately five to six hours though it may remain in the bloodstream for up to 24 hours [5, 7]. While passive environmental exposures may contribute in a small degree to increased breath CO concentrations, a reasonable cut-off level of 3–7 parts per million (ppm) should effectively lower false-positive results [8–10]. 

Previous research on nicotine, a cholinergic nicotinic agonist seen as a main ingredient in cigarettes has focused on the role of cholinergic nicotinic activity in the brain [11]. Cholinergic neurotransmitter systems are responsible for the release of the neurotransmitter acetylcholine, and are thought to be important in the regulation of forebrain activity and arousal [12]. Cholinergic pathways are involved in selective attention and information processing in humans, and disruptions of these pathways can affect the efficiency with which humans receive and process auditory and other sensory information. Prenatal and neonatal exposure to nicotine from smoking has shown to alter or diminish the functioning of the cortical nicotinic acetylcholine receptors leading to long-term negative effects on auditory-cognitive functions in adult rats [13, 14]. Prenatal and adolescent exposure to tobacco smoking was found to exert gender-specific reduction in cortical cholinergic markers and deleterious effects on auditory attention [15]. 

Smoking increases the human body's need for oxygen while reducing the amount of available oxygen capable of reaching the bloodstream; thus, the amount of oxygenated blood that can reach the vital organs is decreased [16]. Cigarette smoking has been shown to be highly associated with the development of hearing loss [17, 18]. The cochlea is highly susceptible to smoking, and otoacoustic emission measures (distortion product otoacoustic emissions (DPOAE) and transient otoacoustic emissions (TEOAE)) have been used to provide early indications of cochlear dysfunction. Young adult smokers with normal hearing were found to have significantly reduced DPOAEs compared to their normal counterparts [19, 20].

Nicotine is known to cause dysynchrony of cortical activity in electroencephalographic (EEG) experiments [21–23]. The neurotransmitter found at the synaptic junctions of the medial olivocochlear bundle (MOCB) neurons and the outer hair cells (OHCs) is a nicotinic cholinergic neurotransmitter especially susceptible to harmful effects of nicotine exposure [24]. Cholinergic system innervation of brainstem and midbrain regions is also well documented, and it seems likely that nicotine exposure would affect some aspects of the auditory brainstem responses (ABRs) [25]. However, there is a wide variation among studies, and the effects of smoking/nicotine on ABRs have shown inconsistent results in terms of increase or decrease of ABR parameters [25]. Some of these differences can be attributed to subject variables and whether or not the acute or chronic effects of nicotine were evaluated. Based on the existing research, it is likely that the effects of cigarette smoking on auditory sensitivity and function can be documented with clinical tests. 

Research involving cigarette smokers and auditory function has focused on adults over 25 years of age. These adults have been exposed to greater levels of environmental toxins and noise, which contribute to the auditory deficit and aging effects. According to CDC, 18–24 year-old use tobacco products more than any other age group [1]. Moreover, these younger adults have a history of smoking during their adolescent years. Research findings have shown that exposure to tobacco smoke during prenatal as well as during adolescent years exerts deleterious effects on the neural circuitry supporting auditory attention [14, 15]. Yet there is limited data on the early effects of chronic smoking on the auditory system in young adults. 

Early effects of cigarette smoking on the auditory system must be identified before audiometric pure tone testing shows a loss of hearing sensitivity so that appropriate actions may be discussed with these individuals regarding hearing conservation. The purpose of this study was to identify if there is a relationship between the subjects' auditory measures and their breath CO level using Best-Subsets Linear Regression Analysis procedures. CO was selected because it is one of the main ingredients in smoke cigarettes and is measurable using a simple tool. In this study, auditory acuity and auditory signal processing were evaluated using an auditory test battery consisting of behavioral and electrophysiologic test measures. Results from the test battery may reveal information on peripheral and central neural auditory pathway measures in smokers who would otherwise exhibit normal hearing sensitivity. These measures may then be used as sensitive tools in the early detection of hearing loss associated with cigarette smoking.

## 2. Material and Methods

CO measures are shown to provide an accurate assessment of cigarette smoking with a specificity of 98% in relation to smoking status. Hence, this study was designed to identify any relationship that may exist between auditory measures and CO levels in young male chronic smokers using multiple regression analysis. An important aspect of any multiple regression analysis is the determination of the relative importance of the various predictors. Researchers frequently use regression models to predict outcomes, but a balance must be maintained between including too many variables and model parsimony. Omitting important prognostic factors results in a systematic misestimation of the regression coefficients and biased prediction, but including too many predictors will result in loss of precision in the estimation of regression coefficients.

### 2.1. Subjects

This study was approved by the University of North Texas Institutional Review Board. Sixteen adult male smokers with no history of hearing loss took part in this study. All subjects were healthy: 18–24 year-old male chronic smokers with a history of smoking for an average of 4 years, with a mean use of 17 cigarettes per day. All subjects underwent a thorough screening process, wherein they were questioned regarding their general health, hearing loss, psychological or neurological problems and smoking habits.

### 2.2. Procedures

Detailed case history information was obtained from all subjects. The standard clinical case history form from the University of North Texas Hearing Clinic was used in addition to a set of questions designed to examine smoking history. This additional set of questions elicited responses including number of cigarettes smoked per day and age of smoking commenced. Any change in smoking habits in the recent past was also noted. All subjects were tested within an hour of smoking a cigarette. 

A CO monitor Micro 4 Smokerlyzer was used to measure breath CO. All subjects were required to hold their breath for 15 seconds and to exhale slowly into the mouthpiece of the Smokerlyzer. The Micro 4 displayed the CO reading in a few seconds. A new mouthpiece was used for each subject. Each reading was repeated twice for consistency. The CO level in ppm was used as the outcome measure. 

Following case history information, an otoscopic examination was conducted to rule out peripheral abnormalities. A battery consisting of several audiological tests was administered, and the order of the tests was randomized to minimize test order effect. Pure tone audiometry using the modified Hughson-Westlake procedure was conducted in a double-walled sound-treated room using an audiometer that was calibrated to the American National Standards Institute (1989) specifications [26]. Octave frequencies from 250 Hz to 8000 Hz were tested. Pure tone average was obtained by averaging hearing thresholds at 500, 1000, and 2000 Hz. Using a calibrated Grason-Stadler GSI-33 middle ear analyzer, tympanograms were obtained. Acoustic reflex thresholds (ARTs) were measured ipsilaterally and contralaterally at 500, 1000, and 2000 Hz. The rationale for using pure tone audiometry and immittance audiometry prior to other tests was to rule out hearing loss and peripheral disorders (outer and/or middle ear problems). 

Auditory evoked potentials (AEPs), specifically auditory brainstem responses (ABRs) and middle latency responses (MLRs), were obtained in order to evaluate the performance of the retrocochlear and central auditory systems. AEPs were recorded for right and left ear stimulation separately, using the ICS (ICS Medical, CHARTR Diagnostics System, Model MCU-90, Schaumburg, Ill, USA). The stimuli were presented through disposable ear tips (ER3). Gold cup electrodes were placed on the high forehead (active electrode), right and left earlobes (reference electrodes), and nasion (ground electrode). The absolute impedances were kept below 5000 Ω in all subjects, while the interelectrode impedance differences were within 1000 Ω of each other. Every run was repeated at least twice to ensure repeatability. 

ABRs were recorded using rarefaction clicks with a presentation rate of 21.1 clicks/sec for intensities ranging from 40 to 80 dB nHL in 10 dB steps introduced in a random order. The responses were amplified 100 000 times and filtered using a bandpass filter that was set between 100 and 3000 Hz. A minimum of 1500 sweeps were averaged, with a time window set to 10 milliseconds. The analysis focused on the most prominent component of the ABR wave, peak V. Latencies and peak-to-trough amplitudes for ABR peak V were measured in all subjects. MLRs were also recorded using rarefaction clicks at intensity levels ranging from 40 to 80 dB nHL. The presentation rate was 9.1 clicks/sec. Responses were amplified 50 000 times and filtered using a 10-to-100 Hz band-pass filter setting. The responses were averaged for a minimum of 500 runs with the time window set to 50 milliseconds. Absolute latencies and amplitudes were measured for MLR peaks Na and Pa. ABR and MLR peak latencies and amplitudes were identified and measured independently by two individuals knowledgeable in peak identification methods and compared to the normative data collected and published by K. Gopal [27].

OAE tests were conducted to obtain information on the status of the outer hair cells in the cochlea. A Madsen Capella cochlear emissions analyzer was used to measure spontaneous otoacoustic emissions, transient otoacoustic emissions (TEOAEs), and distortion-product otoacoustic emissions (DPOAE). TEOAEs were collected for a minimum of 600 runs using nonlinear 80 microseconds clicks at 84 dB SPL at 1, 1.4, 2, 2.8, and 4 k Hz. The test was repeated twice for reliability. DPOAEs were recorded with the DP-Gram procedure. The 2f1-f2 DPOAEs were recorded at a single level of f1 = 65 dB SPL and f2 = 55 dB SPL. The f2/f1 ratio was held constant at 1.22 and responses were measured at 1, 1.4, 2, 2.8, and 4 k Hz.

## 3. Statistical Methods

### 3.1. Software

The base R statistical computing environment (version 2.6.1) and additional packages (http://www.cran.r-project.org/src/contrib/PACKAGES) were used for data manipulation and statistical modeling. The principal supporting packages used were (1)* leaps* (best subsets regression), (2) *relaimpo* (relative importance effects), (3) HH (support software for *s*tatistical analysis and data display).

### 3.2. General Modeling Strategies

Using the data from 16 adult male smokers, 24 audiological measures were used to develop a predictive model for the CO level (outcome measure). Ordinary least squares (OLS) estimation procedures were used with best-subsets selection and bootstrapped stepwise variable selection procedures [28, 29] to develop an optimally predictive multiple linear regression model. The predictors for the final chosen model were based on the restriction that only five variables would be retained giving approximately a 3 to 1 case/variable ratio due to the small sample size. Because the sample size was small in comparison to the number of variables used, the final model was selected to account for biased fit indices. Consequently, a bootstrapped stepwise procedure, using the Akaike information criterion (AIC) model fit index, was used to select the best 5-variable model that was reproduced (validated) with 500 resampled datasets. The 5-variable model that occurred the largest percentage of times out of the 500 resampled datasets was selected [29]. This bootstrap stepwise AIC model was then compared to the best 5-variable, best-subsets linear regression, and the final best-subsets model was based on adjusted R-squared and AIC indices. Theoretical considerations would suggest that these two variable selection methods should be similar in the final models that are selected. Furthermore, a convergent validity check was conducted for these two methods.

### 3.3. Methods Used to Interpret the Importance of Regression Predictor Effects

The present study was also concerned about appropriate accounting for the intercorrelations between the predictor variables (X1,…, Xp). So an effect size measure (lmg statistic) was calculated for predictors that can be considered unambiguous with regard to variable importance [30]. The lmg statistic can be interpreted as the average squared semipartial correlation coefficient for a predictor, where the averaging takes place over all possible orderings of that predictor variable within the set of all predictors under scrutiny. Additionally, it is presumed that interpretation of predictor effects is best conducted by comparing the lmg statistic with appropriate confidence intervals based on bootstrap confidence intervals [29]. The term “bootstrap” refers to a computer-intensive resampling method for estimating the variability of statistical quantities and for setting confidence regions for parameters. This study used bootstrap procedures to estimate confidence intervals (precision) for regression predictor weights (betacoefficients) and importance statistics (lmg statistic). This was accomplished by boot-strapping parameter estimates and model fit indices for linear regression (i.e., linear regression based on stepwise AIC variable selection). Bootstrapping has been shown to be effective with sample sizes as little as *N* = 10 [31]. 

## 4. Results

The mean and standard deviation CO level in the subject group was 22.7 ± 8.6 ppm. The optimal cut-off point of exhaled CO for detecting smoking is 7 ppm [9]. Based on established clinical audiological norms, all 16 subjects had normal hearing thresholds, type A tympanograms, normal acoustic reflex thresholds, normal otoacoutsic emissions, and normal ABR measures. It must be noted that although all of the test measures were within the normative data, the level of performance varied among the subjects. From the test battery, 24 different auditory measures were identified as possible predictor variables, and are shown in Table 1. The bootstrap stepwise AIC model and the best-subsets linear regression model chose the same five predictors from the 24 original variables. All five predictors chosen were electrophysiologic measures, and are shown in Table 2. The amplitude measures for the ABR peak V and DPOAE outcomes account completely for the five selected predictors in the model. Qualitatively, the directional associations of the amplitude measures for right ear ABR peak V at 80 dB nHL, and 40 dB nHL (RTABRPV80A and RTABRPV40A), indicated a positive association with CO levels (column 2 in Table 2). These positive predictor coefficients imply that as CO level increases, the amplitude measures increased correspondingly across subjects. Similarly, the DPOAE measures for the right ear (RTDP) was also positively associated with CO levels, that is, increases in DPOAEs were associated with increases in CO measurement levels. In contrast to the right ear measures, the left ear ABR peak V amplitude (LTABRPV80A) as well as left ear DPOAE measure (LTDP) decreased with increase in CO level. 

Noting directional relationships between predictors and outcomes allows for a qualitative view of the data. However, a more precise view of the data can emerge by accounting for the magnitudes (strength) in the predictor-outcome relationships. In the present study, the best-subsets model with five predictors foreseeing CO levels, resulted in effect sizes (the lmg statistic: these are estimated R-squared values) which, relative to the total sum of 1.00 (see Note 2 Table 2), ranged from the largest value of 0.39 to the smallest value of 0.06. The majority of these predictors would qualify as predictors with “large to medium” effect sizes (39%, 30%, 15%, 11%), except for the right ear measure of the distortion product (RTDP) which might be characterized as having a smaller effect size (6%). Moreover, the predictors as a set accounted for 75% (predicted variance) of the total variance (predicted plus residual variance) in CO levels—a very large total effect size for the model. As previously noted, the predictors are each accounting for independent variance (nonoverlapping variance) in CO levels, and these independent relative contributions sum to 1.00 and account for 75% of the total variance in CO levels (the model fit value: R-squared). In other words, the predictors, as a set, independently account (nonoverlapping) for 100% of 75% variance predicted in CO levels. Having the lmg statistic normalized so that the values sum to 1 aids in judging the relative contributions in predicting CO levels. 

Ranking the predictors beta coefficients will be implicit in any magnitude comparisons of these beta coefficients. However, as discussed previously, correlations among the predictors can render the direct comparisons of model coefficients as problematic. The value of the lmg statistic as an effect size is that it allows the unambiguous ranking of predictor importance for the purposes of model interpretation. The ranking of the estimated predictor effects for the present study are presented in the last column of Table 2. The RTABRPV40A measure accounted for the largest variance in CO levels—approximately 40% of the 75% variance accounted for in CO levels. The second largest predictor, LTABRPV80A, accounted for 30% of the variance in the 75% variance accounted for in CO levels. The third largest predictor, LTDP, accounted for 15% of the variance in the 75% variance accounted for in CO levels. These three predictors and their corresponding effect sizes account for the bulk (85%) of the model fit index R squared (0.75). In effect, these three variables are mostly *what* the large model effect size (0.75) is composed of. 

While point estimates of effect size or importance measures do provide quantitative information about data patterns, it is necessary to accompany these point estimates (e.g., estimated lmg = .39) with estimates of precision. Hence, confidence intervals can be used to obtain estimates of how sampling variability will affect future point estimates with repeated sampling. Estimated confidence intervals will provide a range whose upper and lower points give “best” and “worst” case estimates for future data samples. Bootstrap methods can provide nonparametric confidence intervals for mathematically difficult or intractable test statistics (e.g., eigen-values, R-squared values, quantile estimates). Table 3 provides nonparametric bootstrap 95% confidence intervals for each point estimate of the lmg statistic for the predictors of our selected model. These intervals represent 95% of the variation in the lmg statistic across sampling with replacement from our original dataset, and provide some idea of how sampling variability will affect potential values of the lmg statistic. This information is useful in two ways: (1) the lower bound of the interval gives us a conservative estimate of how much independent variation in the outcome variable (CO) is represented by that predictor; (2) for purposes of ranking the relative importance in predicting CO, intervals that do not overlap, or that overlap minimally, provide stronger evidence that our point estimates of lmg in our sample, represent different quantities measured on the “superset” or the population of interest. 

## 5. Discussion

Numerous studies have identified the exposure to cigarette smoking as a risk factor for hearing loss. This study, unlike other studies, was designed to identify the preclinical auditory predictors in young adult smokers who still preserve normal hearing thresholds. Twenty-four auditory test measures (predictor variables) were collected on 16 subjects, along with their CO level (outcome variable). The joint relationship between the predictor variables and the outcome variable was estimated using bootstrapped stepwise AIC and best-subset linear regression. Additionally, nonparametric bootstrap confidence intervals were obtained for regression parameters and the lmg statistic. 

The 24 predictor variables collected from audiologic testing included behavioral measures as well as electrophysiologic measures. Out of the 24 predictor variables, the predictive model identified five variables that were recognized as significant predictors in all bootstrap samples. All five significant variables (Table 2) were found to be electrophysiologic measures. No correlations were found between CO level and pure tone averages or between CO level and ipsilateral/contralateral ARTs. 

The results indicated that DPOAEs, but not TEOAEs, were significant predictor variables. Otoacoustic emissions results indicated larger DPOAE amplitudes compared to TEOAE amplitudes in this group, despite the fact that the noise floor levels were comparable across the frequency range for the two tests. Although this could contribute to the differences in the observed outcome, DPOAEs have, however, been used often as an index of cochlear integrity [19], signifying the greater sensitivity of DPOAEs to cochlear damage. Of the 14 ABR and MLR peak latency and amplitude values used in the study, ABR peak V amplitude measures for 80 dB nHL in right and left ears, as well as for 40 dB nHL in the right ear were found to be significant predictors. Further, it was observed that within the ABR peak V amplitudes and the DPOAEs that made it to the predictor list, an increase in the amplitude correlated with an increase in CO levels in the right ear. The left ear showed the opposite relationship, that is, a decrease in the amplitude correlated with an increase in the CO level. These findings indicate enhancement of activity in the right ear and suppression of activity in the left ear with higher levels of CO. This could be attributed to elevated cochlear blood flow following increased CO in the blood supply to compensate for tissue hypoxia at low concentrations [32], in the dominant ear. All but one subject in this study showed right ear advantage (based on a screening test of auditory processing). The stimulatory effects on the projection pathways with input to the right ear may have resulted in increased cortical and subcortical excitability. On the other hand, an inhibitory effect of the projection pathways with input to the left ear may have resulted in decreased excitability. Yet another explanation for this observation could be the increased nicotine level in the smokers, which can enhance suppression of the MOCB, and consequently increase activity of the neural fibers leading to increased amplitude measures and increased DP levels in the dominant right ear. Moreover, without longitudinal data, transient effects cannot be ruled out in the right and left ear performance differences. 

It is important to note that with large sample sizes and all other things being equal, thus confidence intervals shrink. Estimates of lmg that are larger than 10% will, with large sample sizes, reject a null hypothesis test of no difference. It is well known that observed *P*- values for test statistics decrease as a function of sample size with the population effect held constant (the power of the statistical test converges to 1 as n increases without bound). That is to say, ranked predictors A-D would, with larger sample sizes, be viewed (in a probabilistic sense) as measuring nonoverlapping entities in the population. This study, being exploratory in nature, suffers from small sample sizes. Hypothesis tests based on small sample sizes (even with large effect sizes) should be viewed with caution. Despite this limitation, the present study provides point estimates that range from small to large with the majority of the point estimates of lmg being larger than 10 percent (4/5 of the estimates). For this reason, we are inclined to give more weight to the magnitude of the lmg statistic rather than the width of the confidence interval in our interpretation of the results. The confidence intervals for the ranked predictors based on lmg are provided as additional information to readers who are more comfortable interpreting hypothesis tests.

One of the limitations of this study is that only male subjects were used; consequently, generalization cannot be made across genders. Only males were selected in this pilot study to control for possible hormonal differences. Secondly, smoking habits were determined solely on subjects' self reports although CO levels in all subjects indicated higher levels than what is normally seen in nonsmokers. Furthermore, CO levels were used as the outcome variable, rather than using direct blood-nicotine levels. Even though nicotine is the main ingredient in cigarattes, there are many toxic substances that are released when tobacco leaves burn, including CO. Due to budgetary issues, the CO level assessment was adopted in this study.

## 6. Conclusions

The prevalence of smoking among college-age individuals (18–24 years) continues to increase, regardless of previous and emerging information about the health risks. The clinical significance of this research is that in young male chronic smokers with normal auditory measures, certain electrophysiologic measures showed a significant relationship to CO level. This implies that the effect of smoking can be identified and measured using electrophysiologic measures even when hearing sensitivity is well within normal limits. 

## Figures and Tables

**Table 1:** The 24 test measures selected as possible predictors from the audiological test battery (left column) and their abbreviations (right column). Note. Pure tone average was obtained by averaging hearing thresholds at 500, 1000, and 2000 Hz.

Test measure	Abbreviations
Right ear pure tone average	RTPTA
Left ear pure tone average	LTPTA
Right ear ART ipsilateral condition	RTARTIPS
Left ear ART ipsilateral condition	LTARTIPS
Right ear ART contralateral condition	RTARTCON
Left ear ART contralateral condition	LTARTCON
Right ear Transient Emissions	RTTE
Left ear Transient Emissions	LTTE
Right ear Distortion Products	RTDP
Left ear Distortion Products	LTDP
Right ear ABR peak V latency at 80 dB nHL	RTABRPV80L
Left ear ABR peak V latency at 80 dB nHL	LTABRPV80L
Right ear ABR peak V amplitude at 80 dB nHL	RTABRPV80A
Left ear ABR peak V amplitude at 80 dB nHL	LTABRPV80A
Right ear ABR peak V amplitude at 40 dB nHL	RTABRPV40A
Left ear ABR peak V amplitude at 40 dB nHL	LTABRPV40A
Right ear MLR peak Na amplitude at 80 dB nHL	RTMLRNA80A
Left ear MLR peak Na amplitude at 80 dB nHL	LTMLRNA80A
Right ear MLR Peak Na amplitude at 40 dB nHL	RTMLRNA40A
Left ear MLR Peak Na amplitude at 40 dB nHL	LTMLRNA40A
Right ear MLR peak Pa amplitude at 80 dB nHL	RTMLRPA80A
Left ear MLR peak Pa amplitude at 80 dB nHL	LTMLRPA80A
Right ear MLR Peak Pa amplitude at 40 dB nHL	RTMLRPA40A
Left ear MLR Peak Pa amplitude at 40 dB nHL	LTMLRPA40A

**Table 2:** Best-Subsets Linear Regression Coefficients of five optimal predictors (selected from 24 original predictors) of carbon monoxide level based on *N* = 16 cases.

Predictor	Estimate	StandardError	*t* value	Pr(>|*t*|)	lmg statistic with ranking of importance in predicting outcome measured
Intercept	32.3172	1.8146	17.809	6.65e-09***	
RTABRPV80A	11.8526	4.5424	2.609	0.026071*	0.1053425-D
LTABRPV80A	−61.7999	4.8624	−12.710	1.70e-07***	0.2971096-B
RTABRPV40A	92.3103	5.6452	16.352	1.52e-08***	0.3937055-A
RTDP	0.6637	0.1181	5.618	0.000222***	0.0572460-E
LTDP	−1.4257	0.1549	−9.203	3.38e-06***	0.1465964-C

Note 1: Total Multiple R-squared: .7449 (74.49 % variance accounted for in CO by the predictors). Significance of probability indicated by: 0 – “***” 0.001 – “**” 0.01 – “*”.

Note 2: All 74.5% of variance in the outcome variable (CO) is accounted for by 5 independent (non overlapping) sources of variance (optimal predictor set). Consequently, the total sum for lmg across predictors adds to 1.0.

**Table 3:** Relative importance 95% confidence intervals (95% CI).

		95% CI	
rank of the predictor based on the size of lmg	lmg point estimate	lower	upper
(D) RTABRPVA80	0.1053	0.0531	0.2071
(B) LTABRPVA80	0.2971	0.1516	0.4898
(A) RTABRV40	0.3937	0.1487	0.6176
(E) RTDP	0.0572	0.0188	0.1906
(C) LTDP	0.1466	0.0474	0.3054

Note. Letters indicate the ranked predictor that is covered by the lower and upper bootstrap confidence intervals for the lmg statistic.
